# *Scedosporium apiospermum*-induced brain abscess leading to rapid mortality in an immunocompetent adult male from Uttarakhand, India

**DOI:** 10.22034/cmm.2025.345248.1587

**Published:** 2025-02-01

**Authors:** Minakshi Singh, Priyal Anand, Sowjanya Perumalla, Amber Prasad

**Affiliations:** Department of Microbiology, All India Institute of Medical Sciences, Rishikesh, India

**Keywords:** Brain abscess, Fungal brain abscess, *Monosporium apiospermum*, *Scedosporium apiospermum*

## Abstract

**Background and Purpose:**

*Scedosporium apiospermum*, a soil-dwelling fungus, is typically associated with localized infections, such as skin infections and osteomyelitis. However, it can also cause invasive central nervous system infections, including brain abscesses, particularly in immunocompromised individuals. Such infections are rare in immunocompetent individuals and often occur following trauma or environmental exposure. This report aimed to present a case of a fatal *S. apiospermum* brain abscess in an immunocompetent adult male, highlighting diagnostic and management challenges.

**Case presentation:**

A 48-year-old immunocompetent male presented with a three-day history of persistent holo-cranial headache and left-sided weakness. Twenty days earlier, the patient had fallen into a sewer, likely exposing him to fungal pathogens. Initial imaging revealed a large right frontal intracranial lesion. Surgical resection of the abscess was performed, and antifungal therapy with voriconazole was initiated.

Intraoperative findings revealed a thick, non-vascular abscess capsule containing yellow pus. Postoperative KOH mount confirmed fungal elements (hyaline septate hyphae). Despite aggressive management in the intensive care unit, including antifungal therapy, antibiotics, and supportive care, the patient developed septic shock and succumbed to cardiac arrest within 48 h of surgery.

**Conclusion:**

This case underscores the rapid progression and severity of *S. apiospermum* infections in immunocompetent individuals, even with early surgical and medical intervention. It emphasizes the need for heightened clinical suspicion in cases involving trauma with potential environmental exposure. Prompt diagnosis, effective antifungal therapy, and multidisciplinary management are essential to improve outcomes in such cases.

## Introduction

At least 5 species of *Scedosporium* (*S. apiospermum*, *S. boydii*, *S. aurantiacum*, *S. dehoogii*, and *S. minutisporum*) can cause human infections [ 1 ]. *Scedosporium* is a genus of fungi that includes *S. apiospermum* and *S. prolificans*. These fungi are commonly found in soil, polluted water, and sewage, and infections caused by them are collectively referred to as Scedosporiosis [ 2 ]. Although *S. apiospermum*, formerly known as *Monosporium apiospermum*, is typically associated with various clinical manifestations such as skin infections and osteomyelitis, it has a predilection for causing central nervous system (CNS) infections, particularly brain abscesses [ 3 , 4 ].

Brain abscesses are generally more frequent in immunocompromised individuals; however, they can also occur in otherwise healthy individuals, particularly those with a history of environmental exposure, such as trauma or immersion in contaminated water [ 5 ]. CNS infections caused by *S. apiospermum* are known for their severe progression and high mortality rate, which has been reported to be as high as 74% [ 6 ]. A systematic review by Firoozeh Kerman et al. evaluated thirty-eight studies involving 41 patients from January 1, 2007, to April 20, 2022, and found an overall mortality rate of 51.2% due to *Scedosporium* infections [ 7 ]. Previous studies have shown that the mortality rate can reach up to 65%-100%, once *Scedosporium* disseminates systematically or invades the brain [ 8 ].

The diagnosis of Scedosporiosis presents significant challenges due to its clinical and microbiological similarities with other fungal infections. Accurate identification relies heavily on microbiological culture and histopathological examination, making early and precise diagnosis crucial for effective treatment. Treatment typically involves a combination of surgical intervention and antifungal therapy, with voriconazole being the preferred drug [ 8 , 9 ]. This report describes a case of a rapidly fatal brain abscess caused by *S. apiospermum* in an immunocompetent adult male, emphasizing the urgent need for prompt diagnosis and intervention.

## Case Presentation

A 45-year-old male presented with a 3-day history of moderate holocranial headache, which showed mild relief with medication, accompanied by left-sided weakness. He had no episodes of vomiting, seizures, or loss of consciousness. There was no history of diabetes, hypertension, smoking habit, or any other metabolic disease. The patient had fallen into a sewer 20 days prior and was initially managed at another facility before being transferred to the All India Institute of Medical Sciences, Rishikesh, India, for further treatment. Although he could respond to commands by opening his eyes, he was unable to speak. Physical examinations of the pulmonary, cardiovascular, and abdominal systems were unremarkable. 

Initial investigations on day 1 of hospitalization included complete blood count, blood urea, serum sodium, serum potassium, creatinine, liver function tests, and a contrast-enhanced magnetic resonance imaging (MRI) of the brain. The MRI revealed a T2 hyperintense right frontal mass measuring 6.3 × 4.1 × 4.1 cm with post-contrast peripheral enhancement, suggesting a cerebral abscess. Surgical intervention was planned for the following day, and viral markers were tested, and all results were negative.

During the surgery, a right fronto-temporo-parietal craniotomy was performed. The tense dura was opened, revealing a significant brain bulge. Cortisectomy of the right middle frontal gyrus exposed an abscess cavity with a thick, non-vascular capsule containing pale yellow pus. Gross total resection was achieved, and the abscess capsule was removed. The bone flap was stored in the abdomen, and pus was sent for microbiological analysis, including Gram stain, KOH testing, aerobic bacterial culture, fungal culture, Ziehl-Neelsen (ZN) staining, cartridge-based nucleic acid amplification test (CBNAAT) for *Mycobacterium tuberculosis*, and histopathology (Table 1). 

**Table 1:** Key laboratory findings and their significance for the case.

Test	Day 1 Results	Day 2 Results	Comments
Contrast-enhanced MRI of the brain	T2 hyperintense mass (6.3×4.1×4.1 cm) with peripheral enhancement	Not done	Suggestive of cerebral abscess
Hemoglobin	13.7 g/dL	11.3 g/dL	Decreased over 24 h
Total leukocyte count	19.1×10^3^ cells/µL	18.8×10^3^ cells/µL	Slight decrease
Platelet count	173×10^3^/µL	125×10^3^/µL	Decreased over 24 h
Blood urea	74 mg/dL	57 mg/dL	Improvement noted
Serum creatinine	1.28 mg/dL	0.9 mg/dL	Improvement noted
Serum sodium	141 mmol/L	154 mmol/L	Increased
Serum potassium	Within normal limits	Within normal limits	Within normal limits
Liver function tests (SGPT/SGOT)	SGPT: 58 U/L, SGOT: 31 U/L	Not done	Within normal limits
Gram stain (pus from the brain)	No microorganisms observed, abundant pus cells seen	Not done	No bacterial microorganism observed
KOH examination (pus from the brain)	N/A	Hyaline septate hyphae	Indicated fungal presence
Fungal culture (pus from the brain)	N/A	*Scedosporium apiospermum* (after 14 days)	Confirmed fungal pathogen
ZN Stain and CBNAAT for *Mycobacterium tuberculosis* (pus from brain)	Negative	Negative	No evidence of tuberculosis infection
Viral Markers (HCV, HBV, HIV)	Negative	N/A	Confirmed absence of Hepatitis B, Hepatitis C, and HIV infections

MRI: Magnetic resonance imaging, ZN: Ziehl-Neelsen, CBNAAT: cartridge-based nucleic acid amplification test, SGPT: serum glutamate pyruvate transaminase, SGOT: serum glutamic-oxaloacetic transaminase, HCV: hepatitis C, HBV: hepatitis B, HIV: human immunodeficiency virus

Postoperatively, the patient was admitted to the intensive care unit, where his Glasgow Coma Scale declined to E2V1M5, necessitating intubation and ventilatory support. Despite treatment with antibiotics, antifungals, antiepileptics, analgesics, and decongestants, he developed high-grade fever and hypotension, requiring inotropic support. Based on the KOH findings, voriconazole was initiated, but his condition continued to deteriorate, resulting in a cardiac arrest on the third day. Despite resuscitation efforts, he was declared dead. Fungal culture of the pus obtained during surgery confirmed the presence of *Scedosporium apiospermum* after 14 days of incubation. Cause of death was determined to be septic shock resulting from a right frontal fungal abscess due to *Scedosporium* species, with associated mass effect and midline shift.

### Laboratory investigations

Table 1 summarizes the key laboratory findings and their significance for the case. Contrast-enhanced MRI of the brain revealed a T2 hyperintense mass measuring 6.3 × 4.1 × 4.1 cm with peripheral enhancement, suggestive of a cerebral abscess.

**Figure 1:**
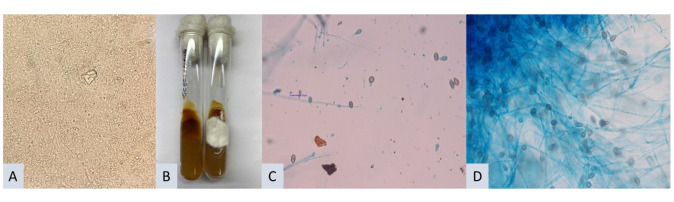
(A) KoH mount of pus sample from brain showing hyaline septate hyphae (40×). (B) Growth on Sabouraud dextrose agar- Grayish black reverse and white cottony growth on the obverse side. (C and D) Lactophenol cotton blue images of the growth showing branching septate hyphae with single-celled ovoid conidia, typical of *Scedosporium apiospermum*

Pus sample from the brain was sent for microbiological analysis. Gram stain of the pus revealed an abundance of pus cells, but no microorganisms. However, the KOH (10%) examination of the pus showed the presence of hyaline, septate hyphae [Figure 1 (A)]. 

No signs of tuberculosis were detected in the pus sample, as acid-fast bacilli were absent in the ZN stain. Additionally, the CBNAAT using the GeneXpert system (Cepheid Inc.) was also negative for *M. tuberculosis*.

The pus sample obtained from the brain abscess was inoculated onto Sabouraud dextrose agar (SDA) and incubated aerobically at 37 °C. After 14 days of aerobic incubation, the culture displayed white, cottony growth on the front, while the reverse side showed a grayish-black coloration [Figure 1 (B)]. A lactophenol cotton blue mount was prepared from the fungal growth, which revealed branching septate hyphae with single-celled, ovoid conidia, characteristic of *S. apiospermum* [Figure 1 (C and D)].

Mould identification was carried out using the VITEK^®^ MS Mould Kit (BioMérieux, France), which provides reagents for protein extraction and inactivation from agar plates for subsequent analysis by the VITEK^®^ MS System (BioMérieux, France). The process involved collecting a 1-2 cm diameter sample of mould from Sabouraud Dextrose Agar (SDA) plates using a sterile swab. The sample was then suspended in 0.9 mL of ethanol (70%), followed by centrifugation. After discarding the supernatant, the pellet was treated with formic acid (70%) and acetonitrile (100%) for protein extraction and inactivation. The resulting protein mixture was spotted on a target slide, dried, and overlaid with the VITEK® MS-CHCA matrix for analysis [ 10 ]. The processed slide was analyzed using the VITEK® MS System (BioMérieux, France) for mould identification based on the mass spectrometric profile, and was identified as *S. apiospermum* [ 13 ].

## Discussion

This case report presents a rapidly fatal brain abscess caused by *S. apiospermum* in an immunocompetent adult male. Although infections due to *S. apiospermum* are commonly seen in immunocompromised individuals, this case underscores that environmental exposure, such as trauma or contamination with soil or water, can serve as a significant risk factor even in healthy individuals. The patient's fall into a sewer 20 days prior to the onset of symptoms likely played a crucial role in facilitating the fungal invasion, highlighting the importance of environmental factors in the pathogenesis of *Scedosporium* infections.

The patient's diagnosis was delayed due to the nonspecific presentation and initial lack of suspicion for a fungal etiology. This is consistent with many previous cases, where initial symptoms often mimic more common bacterial infections, leading to delayed appropriate antifungal treatment [ 2 ]. Unlike some reports where patients had identifiable risk factors such as immunosuppression or history of near-drowning, this patient had no such predispositions apart from a fall into a sewer, which likely introduced the pathogen [ 3 ].

Compared with other reported cases, the age and immunological status of patients have varied widely. The majority of patients with *Scedosporium* infections reported in the literature were immunocompromised [ 2 , 6 ], including those with leukemia, transplant recipients, and individuals on long-term corticosteroid therapy. These patients usually exhibited severe disseminated infections, including brain abscesses, and had a poorer prognosis than immunocompetent individuals.

Previous case reports have documented varying outcomes in *Scedosporium* brain abscesses. Wilson and Kennedy (2013) reported a fatal case of an immunocompetent 69-year-old male with silicosis who developed a brain abscess following environmental exposure [ 3 ]. Similarly, other case reports have documented fatal cases involving immunocompetent individuals with environmental exposure, emphasizing that trauma-related contamination remains a critical risk factor even in the absence of immunosuppression. These cases demonstrate that despite aggressive surgical and medical intervention, *Scedosporium* CNS infections carry a high mortality risk.

Globally, *S. apiospermum* infections affect various age groups and immunological statuses. In a study by Troke et al. (2008) involving 107 patients treated with voriconazole, most were immunocompromised, spanning from children to the elderly, with a high mortality rate, especially among those with CNS involvement [ 8 ]. This study reinforces our case findings, where the patient succumbed to the infection despite prompt surgical and medical intervention due to its aggressive nature and septic shock complications. Review of literature of similar cases of brain abscess by the *Scedosporium* spp. from India and abroad have been depicted in Table 2.

**Table 2:** Review of literature of similar cases of brain abscess due to the *Scedosporium* spp. from India and abroad.

Year	Country	Age/Gender	Possible predisposing condition	Clinical picture	Treatment given	Outcome	References
2008	Turkey	Not specified	Trauma	Rapid progression of neurological symptoms, brain abscess confirmed via imaging and microbiology	Surgical drainage, voriconazole therapy	Worsened	[ 6 ]
2008	Multiple countries	107 patients (varied)	Immunocompromised and some immunocompetent individuals	Fever, headache, focal neurological signs, worsening despite antifungal therapy, often with poor outcomes	Voriconazole therapy (some cases also had surgical intervention)	Often fatal	[ 8 ]
2013	Australia (Sydney)	69 years/male	Exposure to contaminated soil, silicosis	Headache, fever, focal neurological deficits, imaging revealing a large frontal brain abscess	Surgical intervention, voriconazole therapy	Death	[ 3 ]
2012	Multiple countries	Multiple patients	Environmental exposures, such as trauma, soil exposure, and near-drowning	CNS symptoms, including headache, fever, focal neurological deficits, and positive imaging findings	Surgical drainage, voriconazole therapy	Variable	[ 9 ]

CNS: central nervous system, MRI: magnetic resonance imaging

Treatment for *S. apiospermum* infections usually combines surgical intervention and antifungal therapy. In this case, gross total resection (GTR) of the abscess was achieved, which literature supports as crucial for reducing fungal load and improving outcomes [ 10 ]. Voriconazole is the cornerstone of treatment for *S. apiospermum* infections [ 8 ]. Unlike *S. apiospermum*, which demonstrates good susceptibility to voriconazole, *S. prolificans* exhibits notable resistance to most antifungal agents, including voriconazole, making infections with this species particularly challenging to treat [ 11 ]. In contrast, Nesky et al. reported successful treatment of a Pseudallescheria boydii brain abscess with surgical drainage and voriconazole [ 12 ]. Despite starting voriconazole based on KOH findings, the patient's condition worsened. This aligns with literature indicating that while voriconazole is effective, CNS infections have a poor prognosis and high mortality rates even with treatment [ 6 ].

*S. apiospermum* infections, while reported worldwide, rarely lead to brain abscesses. Most cases occur in immunocompromised individuals, yet there are significant instances in immunocompetent patients, particularly after trauma or near-drowning events. The patient's fall into a sewer in this case underscores the potential risk from environmental fungi, suggesting that a more thorough initial evaluation might have been beneficial. Clinicians must maintain a high suspicion for fungal pathogens in similar scenarios. Unfortunately, despite aggressive surgical and medical management, the outcome was fatal, highlighting the urgent need for further research into effective treatment protocols and antifungal therapies. This case reinforces the aggressive nature of *S. apiospermum* infections and the challenges in diagnosing and managing fungal brain abscesses. Previous cases have consistently demonstrated the difficulties in early detection and the limited success of current strategies, emphasizing the need for heightened clinical awareness and advancements in diagnostic techniques to improve patient outcomes.

## Conclusion

This case of *S. apiospermum* brain abscess in an immunocompetent individual highlights the potential for rapid disease progression and the challenges in the diagnosis of fungal CNS infections. Despite the absence of typical risk factors, environmental exposure, such as trauma and contamination from a sewer, likely played a key role in the onset of the infection. Rapid deterioration of the patient, even with aggressive treatment, underscores the high mortality rate associated with these infections.

Early recognition and intervention are critical, as fungal brain abscesses can often mimic more common conditions, leading to diagnostic delays. This case emphasized the need for heightened clinical suspicion in patients with neurological symptoms and a history of environmental exposure, regardless of their immune status. Given the limited success of current antifungal treatments in such severe cases, further research into adjunctive therapies and alternative antifungals is essential. Clinicians should remain vigilant and consider fungal etiologies when managing brain abscesses, especially in cases where environmental factors may be involved. Improved diagnostic methods and treatment strategies are crucial for better patient outcomes.
